# Supplementary figures and images for: Autotrophic growth of Escherichia coli is achieved by a small number of genetic changes
Source: eLife. 2024 Feb 21;12:RP88793. doi: 10.7554/eLife.88793 (PMC10942610; doi:10.7554/eLife.88793)

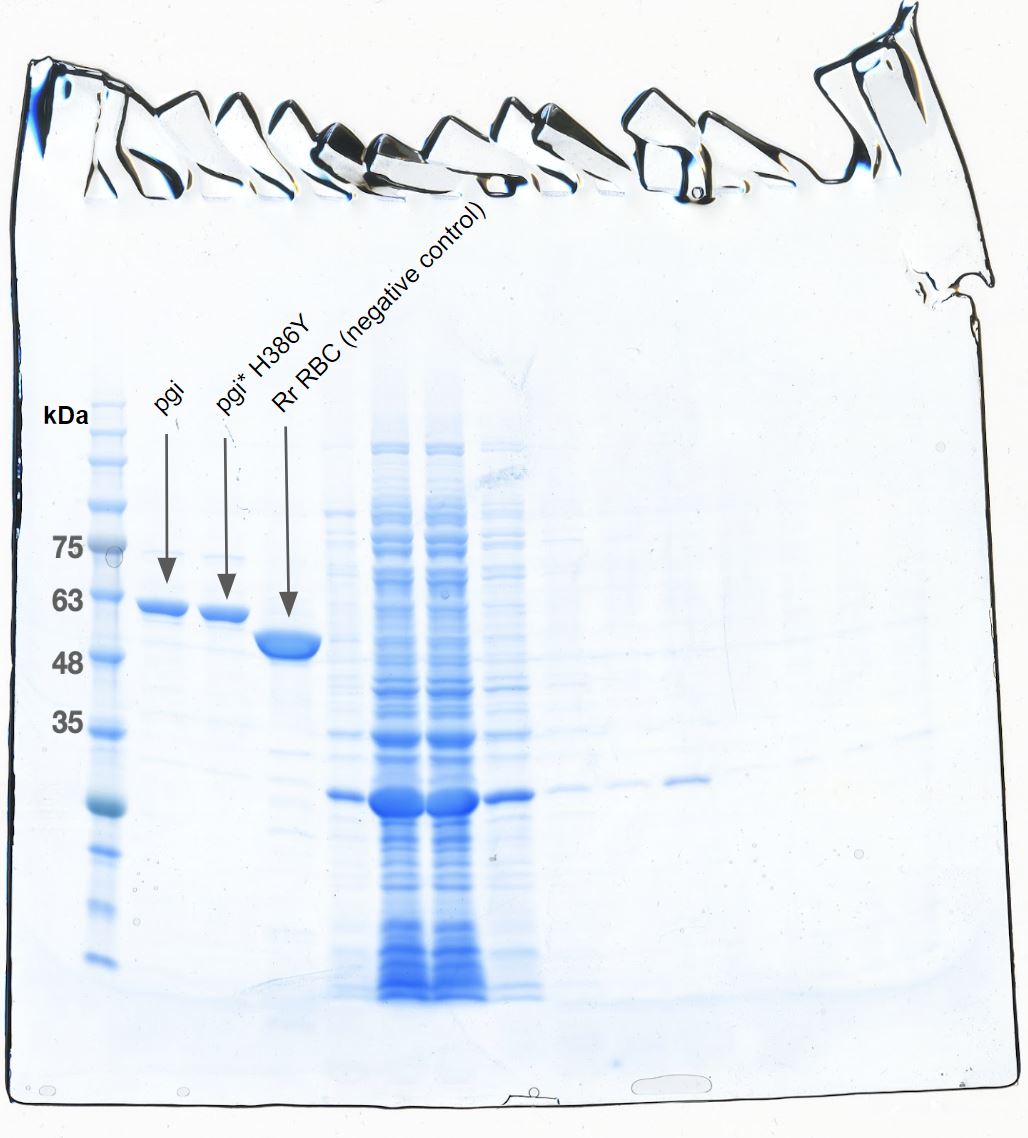

Supplement: Figure 4—source data 1. [file elife-88793-fig4-data1.zip › Gel source edited.JPG]

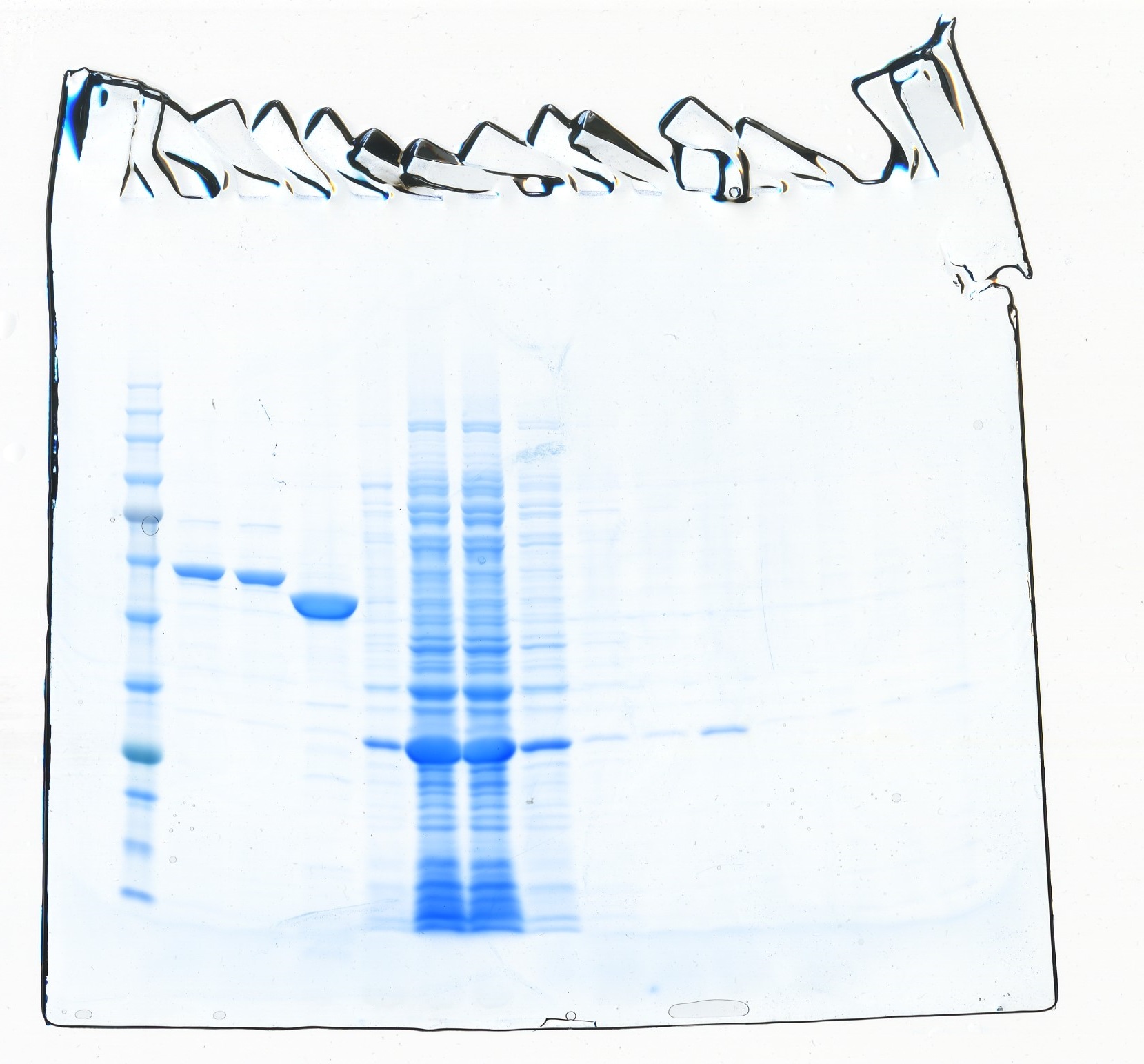

Supplement: Figure 4—source data 1. [file elife-88793-fig4-data1.zip › GEL source.jpg]
